# Changes in temporomandibular joint spaces after arthroscopic disc repositioning: a self-control study

**DOI:** 10.1038/srep45513

**Published:** 2017-03-31

**Authors:** Ying Kai Hu, Ahmed Abdelrehem, Chi Yang, Xie Yi Cai, Qian Yang Xie, Manoj Kumar Sah

**Affiliations:** 1 Department of Oral Surgery, Ninth People’s Hospital, Shanghai Jiao Tong University School of Medicine, Shanghai Key Laboratory of Stomatology, Shanghai, People’s Republic of China; 2 Department of Craniomaxillofacial and Plastic Surgery, Faculty of Dentistry, Alexandria University, Alexandria, Egypt

## Abstract

Disc repositioning is a common procedure for patients with anterior disc displacement (ADD). The purpose of this retrospective record-based study was to evaluate changes in the widths of joint spaces and condylar position changes in patients with unilateral ADD following arthroscopic disc repositioning, with the healthy sides as self-control, using magnetic resonance images (MRI).Widths of anterior, superior, and posterior joint spaces (AS, SS, and PS) were measured. The condylar position was described as anterior, centric or posterior, expressed as 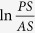. Paired-t test and Chi-square test were used to analyze the data. Fifty-four records conformed to the inclusion criteria (mean age of 21.02 years). Widths of SS and PS increased significantly after surgery (*P* < 0.001) on the operative sides, while joint spaces of healthy sides and AS of operative sides had no significant changes. Dominant location of condyles of operative sides changed from a posterior position to an anterior position, while healthy sides were mostly centric condylar position no matter preoperatively or postoperatively. Therefore, the results of this study indicate that unilateral arthroscopic disc repositioning significantly increases the posterior and superior spaces of the affected joints, without affecting spaces of the healthy sides.

Internal derangement (ID) is a frequent disorder of the temporomandibular joint (TMJ), characterized by an abnormal position of the articular disc, often anteriorly displaced^1^,^2^. Many researchers have associated posterior condylar position in the glenoid fossa with ID, although controversy persists^3^,^4^,^5^,^6^,^7^,^8^,^9^,^10^,^11^,^12^. Gateno *et al*. and Barghan *et al*.^6^,^7^ reported that condyles in patients with ADD were located more posteriorly and superiorly within the fossa than in patients without ADD, and the posterior condylar head displacement was 2.4 times greater than the superior condylar head displacement. Incesu *et al*.^9^ found that posterior condyle position could indicate anterior disc displacement (ADD). However, others have suggested a posterior condylar displacement is a poor predictor for diagnosing ADD, because condylar position has wide variations in asymptomatic volunteers^11^,^12^. But most of the studies included joints with no signs of temporomandibular joint disorders (TMD) as normal samples based only on radiographic and chair-side examinations, leaving the possibility of undetected disc displacements. Frequently, the assessment of condyle-fossa relationship was based on comparisons of joint space area ratios or linear distances between the condylar head and the wall of the fossa^6^.

There were several limitations in previous studies. Firstly, many of the studies included joints with no signs of temporomandibular joint disorders (TMD) as normal samples based only on chair-side examinations and imaging such as plain radiographs, computed tomography (CT) or cone-beam CT (CBCT) to evaluate the joint spaces^3^,^4^,^5^,^7^,^8^, which failed to detect the real position of the disc. Therefore, the asymptomatic volunteers might actually have disc displacement, resulting in large variations in “normal” condyles. Besides, few researches implemented a self-control study by which would lead to more accurate results.

Disc repositioning is a common procedure for patients with ADD, to relieve pain and to improve the range of motion. Arthroscopy of the TMJ has been considered a minimally-invasive procedure to treat symptomatic TMJ ID with arthroscopic lysis or lavage^13^,^14^. But the success rate of arthroscopically disc repositioning, measured by magnetic resonance imaging (MRI), have not been satisfactory^15^,^16^. With the new technique of arthroscopic disc repositioning and suturing we developed in 2001, the success rate of disc repositioning was improved to 95.42% for our team, which had been confirmed by postoperative MRI examination^17^. In a previous study, it was reported that malocclusion commonly occurred after TMJ arthroscopic disc repositioning and improved within 28 days after surgery in most patients^18^. However, there are still many unsolved issues to be explored. For instance, what about the changes in joint spaces and condyle-fossa relationship after malocclusion improvement? Since the TMJ is a linkage joint, will unilateral arthroscopic surgery influence the spaces of healthy side?

MRI allows visualization of both hard and soft tissues and is considered the preferred imaging modality for diagnosing disc displacements TMD^9^,^19^. The purpose of this study was to evaluate changes in the widths of joint spaces and condylar position changes in patients with unilateral ADD following arthroscopic disc repositioning, with the healthy sides as self-control. The investigators hypothesized that the posterior and superior space of the affected sides would increase significantly after surgery, resulting in condyle changing from a posterior position to a centric or anterior position, but without influencing the joint spaces of the healthy sides.

## Materials and Methods

### Patients

This retrospective study included a series of MRIs from patients who underwent unilateral arthroscopic disc repositioning surgeries in the TMJ division of the Department of Oral Surgery in Ninth People’s Hospital, Shanghai Jiao Tong University, School of Medicine. The inclusion criteria were as follows: (1) surgery from April 2014 to April 2015; (2) unilateral anterior disc displacement of TMJ (Wilkes stage II–IV); (3) preoperative TMJ MRI; (4) follow-up MRI at least 1 months after the surgery; (5) no treatment before surgery and only wore soft splint postoperatively. The exclusion criteria were: (1) orthodontic treatment or changing the soft appliance to any other type of appliance during follow-up period; (2) history of infection, maxillofacial injuries, or congenital, developmental and system disorders; (3) poor image quality of MRI, which influenced quantitative measurement.

The study followed the tenets of the Declaration of Helsinki, with approval of the Independent Ethics Committee of Ninth People’s Hospital, Shanghai Jiao Tong University, School of Medicine. Informed consent agreement was obtained from all participants.

### Study variables

The predictor variable was healthy side versus operative side; changes in widths of joint spaces (superior, anterior and posterior) and condylar position after surgery were set as outcome variables. Other variables included gender, age, Wilkes stage and follow-up period.

### Surgical technique

The arthroscopic procedures were all performed by one senior surgeon (Y. C.), with the patient under local anesthesia. A 2.3 mm arthroscope, including an image printer and a video surveillance system (Stryker, San Jose, CA) with a 2.8 mm outer protective cannula, was utilized^20^. The procedures for disc repositioning included anterior release, disc reduction and suturing at the posterior margin of the disc. Usually, overcorrection of the disc was achieved to avoid relapse. The details of the technique have been reported previously^20^.

### Acquisition of MRI

MRI scans were acquired by a 1.5-Tesla imager (Signa; General Electric, Milwaukee, WI) with bilateral 3-inch TMJ surface coil receivers. After obtaining an axial localizer image, sagittal and coronal T1-weighted spin echo sequences in the intercuspal position, and T2-weighted spin echo sequences in the open mouth position, were scanned. MRI images were taken perpendicular (sagittal images) and parallel (coronal images) to the horizontal long axis of the condyle. Detail parameters about the TMJ MRI scans were reported in our previous study^17^,^21^.

### Evaluation of MRI

Linear measurements of TMJ space between the condyle and the fossa were made on the sagittal MRI scan (usually the central slice), which were measured by 2 experienced oral and maxillofacial surgeons (H. Y. K. and A. A.) and remeasured at a 2-week interval, using MB-Ruler measuring software (Markus Bader, Berlin, Germany) with 0.01 mm accuracy. Four linear measurements were made, and the mean value was used for statistical analysis.

#### Joint space

**Figure 1:**
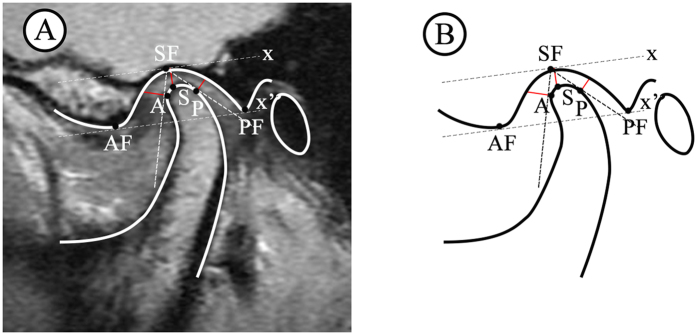
Measurement of joint spaces on MRI. (**A**) In TMJ MRI image, (**B**) Schematic diagram.

Based on the method described in the literature^3^,^9^, a line (x’) drawn between the summits of the post glenoid tubercle (PF) and the articular eminence (AF) was used as the reference plane. The line x was parallel to x’ and tangent to the roof of the glenoid fossa (SF). The distance from the most superior point of the condyle (S) to line x was defined as superior joint space (SS). To measure the anterior joint space (AS) and posterior joint space (PS), lines tangent to the most prominent anterior and posterior aspects of the condyle were made from SF (anterior and posterior tangent points were A and P). Lines perpendicular to the SF/A and SF/P lines were drawn through point A and point P. Anterior joint space and PS were expressed by the distances from point A and point P to the corresponding glenoid fossa (Fig. 1).

#### Condylar position

The position of the condyle was described as anterior, centric or posterior. Centricity was defined as a ±0.25 range on the 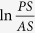 scale. When 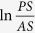 was less than −0.25, the condylar position was posterior within the fossa. While the condylar position was anterior when 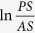 was greater than 0.25^8^.

### Statistical analysis

All the data were entered into a spreadsheet (Excel; Microsoft Inc, Redmond, WA) over the course of the study and analyzed by standard statistical software packages (SPSS, version 17.0, Chicago), and a *P* value of less than 0.05 was considered as statistically significant.

Differences of AS, SS and PS were analyzed by two-tailed paired t-test. Chi-square test was performed to compare the differences of condylar position in two groups before and after surgery, as well as between male and female. Intra- and inter-examiner reliabilities were estimated using intraclass correlation coefficients (ICCs).

## Results

Out of a total of 74 included cases, 12 cases were excluded owing to splint changing and 8 were excluded for poor image quality, leading to 54 cases fulfilling the eligibility criteria. Of these patients, 11 were males. The age of the patients ranged from 11 to 57 years (mean age of 21.02 years). The follow-up period was from 1 to 7 months (mean, 4.17 months). Twenty-seven cases were performed on the right side and 27 on the left side. Five cases (9.3%) were with Wilkes stage II, 42 (77.8%) with stage III, and 7 (12.9%) with stage IV.

ICCs for inter-observer agreement was ranged between 0.80 and 0.95, and intra-observer agreement was ranged between 0.94 and 0.98, showing excellent reliabilities.

**Figure 2:**
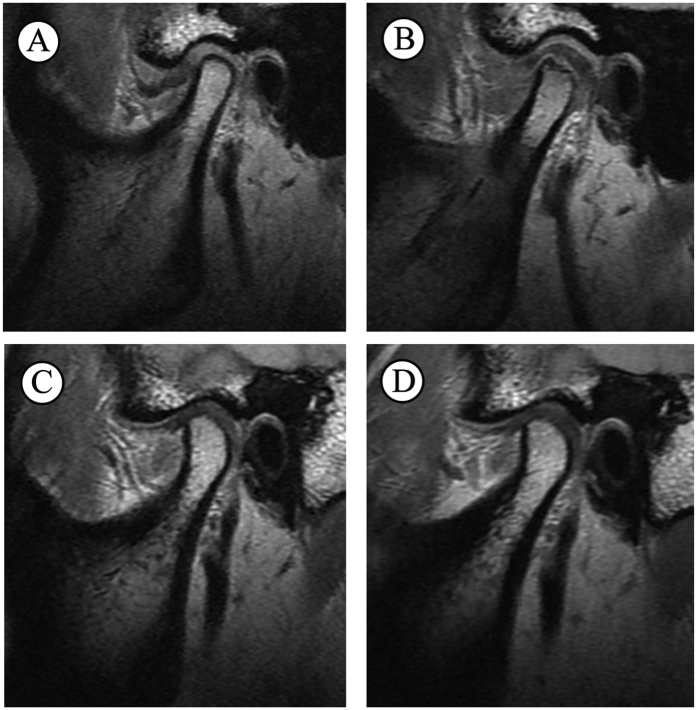
MRI scans of a 42-year-old female patient with ADD on the left side, with the follow-up period of 4 months. (**A**) Operative side preoperatively, (**B**) Operative side postoperatively, (**C**) Healthy side preoperatively, (**D**) Healthy side postoperatively.

As revealed by paired t-test, SS and PS increased significantly after surgery (*P* < 0.001) on the operative sides, while joint spaces of healthy sides and AS of operative sides had no significant changes (Table 1) (Fig. 2). Significant differences were found in preoperative AS and post-operative joint spaces between healthy and operative sides (Table 2). Post-operative joint spaces of operative sides were significantly greater than healthy sides (*P* < 0.001). With regard to condylar position, healthy TMJs showed condyles in a predominantly centric position in the fossa, both preoperatively and postoperatively. Position of the condyles of operative sides changed from posterior position to anterior position. This change in position was significant as shown by chi-square test (Table 3).

There were no significant sex differences in joint spaces (*P* > 0.05), except preoperative SS of operative side and preoperative PS of healthy side (P = 0.007, 0.009, respectively). But no significant differences (*P* > 0.05) demonstrated when compared condylar position between male and female in any two groups.

## Discussion

Arthroscopy of the TMJ was first introduced by Ohnishi^22^, and has been considered a safe and minimally invasive surgical procedure to treat TMJ ID. McCain *et al*.^16^ suggested that malocclusion after disc repositioning might be due to thickening of the retrodiscal tissue, producing an increase in the joint space and concomitant centering of the condyle in the fossa. It has been reported that the incidence of open bite at the posterior teeth on the operative side was 100% on the day of surgery and mostly recovered within 28 days after surgery^18^. In this study, we quantitatively measured the joint spaces changes in unilateral ADD patients who underwent arthroscopic disc repositioning. The results confirmed our hypothesis that the posterior and superior spaces of the affected sides would increase significantly after surgery, leading to condyle changing from a posterior position to an anterior position, but without influencing the joint spaces and condylar positions of the healthy sides.

### Choice of measurement method

TMJ is a joint with complex morphology surrounded by osseous tissues. It is impossible to clinically determine condylar position in the fossa. Thus, various radiographic modalities are used to visualize this position^3^. Conventional radiographic examination would produce superimposition, limiting the accuracy of showing the anatomic characteristics of TMJs^23^. Although computed tomography (CT) or cone-beam CT (CBCT) can delineate the joint structures three-dimensionally with high accuracy, eliminating superimposition^3^,^5^, they fail to confirm disc status. Therefore, so-called “normal” samples in previous studies might have included joints with disc displacement, accounting for the great variability in condylar position^24^. Magnetic resonance imaging makes it possible to view disc displacements, with high diagnostic accuracy for both bone and soft tissue^9^,^19^.

The sagittal slice of MRI allows analysis of joint spaces and the condylar concentricity by comparing the anterior and posterior articular spaces. Linear measurement of the joint spaces, expressed as a logarithmic ratio to the available joint space, was considered a method of choice to describe a radiographic condyle position. It had similar results to area measurements, with merits of high repeatability and easy to use^6^,^8^. Our results proved that quantitative linear measurement on sagittal MRI scan could achieve inter-observer and intra-observer precision.

### Arthroscopic disc repositioning and joint spaces

It has been reported that malocclusion occurred 100% on the day of TMJ arthroscopic disc repositioning and suturing, and remained at a basically stable level after 28 days^18^. Consistent lavage during the operation tended to cause more effusion in the joint space, which might play the most important role for postoperative malocclusion in the first few days after surgery. We collected patients with unilateral ADD who had MRI at least 1 month after arthroscopic surgery, on one hand, we wanted to explore how the surgery influenced joint spaces on normal and affected sides; on the other hand, the interference of joint effusion and malocclusion caused by lavage during the surgery could be minimized.

In the present study, PS and SS of the operative side significantly increased by about 2 mm, while AS changed nonsignificantly. Joint spaces of the healthy side did not show significant change after surgery. Therefore, it is safe to say that arthroscopic disc repositioning can make the condyle go downward and forward, and unilateral surgery would not influence contralateral side. The reasons might lie in several aspects. First, disc repositioning should play the most import role for the increase of joint spaces. Usually, we overcorrected the disc position to prevent relapse of disc displacement, with the squeezed and cumulated bilaminar zone, resulting in immediate enlargement of PS and SS. Despite the posterior band of the disc and bilaminar zone became thinner under the functional movement of the mandible, postoperative PS and SS of the operative side would be still larger than those preoperatively. Furthermore, the disc would become folded, deformed, and thickened after long-term displacement^25^. Thus, the shape of the disc is incompatible with the condyle and fossa when the disc is repositioned. The combined dimension of the condyle/disc often does not fit into the dimensions of the fossa^18^. It might take a long time for the disc to become compatible with the condyle and fossa, which could explain why joint spaces of operative sides were significantly larger than those of healthy sides during an average 4.17 months’ follow-up period.

Kinniburgh *et al*.^26^ found a significant difference in the SS between the sexes using conventional tomography. However, Ikeda and Kawamura^3^ observed that no significant sex difference in AS, SS, or PS with the application of CBCT. Our study indicated no significant sex differences in condylar position and joint spaces, except preoperative SS of operative side and preoperative PS of healthy side. This disaccord might be due to different imaging modalities and sample size.

### Condylar position and ADD

The position of the condyle within the glenoid fossa in patients with TMJ ADD has been controversial. From Table 3, we could observe that ADD was more often (53.7%) associated with posterior condylar position, while the condylar positions of healthy sides were mostly concentric. Our results were in accordance with former studies^6^,^7^,^9^,^27^. The higher prevalence of posterior condylar position in joints with ADD could possibly be explained by the following conditions: (1) the condyle is displaced posteriorly due to the space limitation after disc displacement; (2) the remodeling changes in the condyle or the glenoid fossa, which induced by disc displacement, could change the geometry of the joint; (3) the condyle is originally situated in a more posterior position, thus predisposing the joint to ADD^12^,^28^. According to previous study and our results, it is still not clear whether the posterior condylar position is a consequence or a cause of ADD. However, we could draw a conclusion that the altered joint spaces might indirectly indicate disc displacement, and there was a strong association between TMJ ADD and posterior condylar position.

Occlusal factors might be related to joint morphology^29^, but it was not discussed in our present study. Besides, the follow-up period was relatively short, and the immediate joint spaces changes after surgery were not measured. Moreover, the follow-ups were done at different stages between 1 and 7 months. Consequently, a long-term follow-up study that measures joint spaces at different intervals (0 day, 1, 3, 6, 12 months after surgery) is warranted to explore the changing process of joint spaces and condylar position, as well as when they will become stable.

## Conclusions

This study showed that arthroscopic disc repositioning significantly increases the widths of posterior and superior spaces of the joint, pushing the condyle downward and forward. Unilateral operation does not affect the widths of spaces of the healthy sides, and there was a significant correlation between ADD and posterior condylar position.

## Additional Information

**How to cite this article**: Hu, Y.K. *et al*. Changes in temporomandibular joint spaces after arthroscopic disc repositioning: a self-control study. *Sci. Rep.* **7**, 45513; doi: 10.1038/srep45513 (2017).

**Publisher's note:** Springer Nature remains neutral with regard to jurisdictional claims in published maps and institutional affiliations.

## Figures and Tables

**Table 1:** Changes in joint spaces before and after surgery.

	Pre-operation	Post-operation	t	*P*
	Mean ± SD (mm)	Mean ± SD (mm)		
Healthy side				
Anterior space	2.16 ± 0.60	2.19 ± 0.60	−0.456	0.650
Superior space	2.52 ± 0.74	2.60 ± 0.75	−0.892	0.377
Posterior space	2.15 ± 0.56	2.14 ± 0.64	0.341	0.734
Operative side				
Anterior space	3.00 ± 1.03	3.10 ± 1.08	−0.862	0.393
Superior space	2.64 ± 0.83	4.43 ± 1.29	−10.321	<0.001*
Posterior space	2.34 ± 0.75	4.20 ± 1.11	−13.080	<0.001*

^*^Significant difference.

**Table 2:** Differences of joint spaces between healthy side and operative side.

	Pre-operation			Post-operation		
	Healthy side	Operative side	*P*	Healthy side	Operative side	*P*
Anterior space	2.16 ± 0.60	3.00 ± 1.03	< 0.001*	2.19 ± 0.60	3.10 ± 1.08	<0.001*
Superior space	2.52 ± 0.74	2.64 ± 0.83	0.305	2.60 ± 0.75	4.43 ± 1.29	<0.001*
Posterior space	2.15 ± 0.56	2.34 ± 0.75	0.074	2.14 ± 0.64	4.20 ± 1.11	<0.001*

^*^Significant difference.

**Table 3:** Changes in condylar position.

	Pre-operation N (%)	Post-operation N (%)	χ^2^	*P*
Healthy side				
Anterior	14 (25.9%)	12 (22.2%)	0.209	0.901
Centric	27 (50.0%)	28 (51.9%)		
Posterior	13 (24.1%)	14 (25.9%)		
Operative side				
Anterior	7 (13.0%)	32 (59.3%)	32.995	<0.001*
Centric	18 (33.3%)	17 (31.5%)		
Posterior	29 (53.7%)	5 (9.3%)		
χ^2^	10.229	16.043		
* P*	0.006**	<0.001**		

^*^Significant changes in condylar position on operative sides.

^**^Significant difference in condylar position between healthy side and operative side both pre-operatively and post-operatively.
